# Mobile application prototyping using Artificial Intelligence to support childhood tuberculosis diagnosis

**DOI:** 10.1590/0034-7167-2024-0398

**Published:** 2025-09-08

**Authors:** Katerine Souza Picoli, Flávia Regina Souza Ramos, Denise Maria Guerreiro Vieira da Silva, Bruno da Veiga Thurner, Daniel Souza Sacramento, Irineide Assumpção Antunes, Lucas Lorran Costa de Andrade, Amélia Nunes Sicsú

**Affiliations:** I Universidade do Estado do Amazonas. Manaus, Amazonas, Brazil; II Universidade Federal de Santa Catarina. Florianópolis, Santa Catarina, Brazil; III Universidade Federal de Santa Maria. Santa Maria, Rio Grande do Sul, Brazil; IV Secretaria Municipal de Saúde de Manaus. Manaus, Amazonas, Brazil; V Secretaria do Estado da Saúde do Amazonas. Manaus, Amazonas, Brazil

**Keywords:** Mobile Applications, Artificial Intelligence, Tuberculosis, Pulmonary, Child, Diagnosis, Aplicaciones Móviles, Inteligencia Artificial, Tuberculosis Pulmonar, Niño, Diagnóstico

## Abstract

**Objectives:**

to develop a mobile application prototype using Artificial Intelligence (AI) to predict and support the diagnosis of pulmonary tuberculosis in children – TB Kids.

**Methods:**

technological development research of the prototyping type, based on the Rational Unified Process model and its four stages: conception, elaboration, construction and transition. The development of the TB Kids prototype took place from November 2022 to July 2023.

**Results:**

the TB Kids prototype has features for risk assessment, nutritional assessment, tuberculin skin test, investigation of antibiotic therapy and contacts, interpretation of chest X-rays through AI with risk graph and decision-making, complementary guidance and recording of the clinical picture.

**Conclusions:**

the high-fidelity mobile application prototype has a consistent interface, responding with creativity and innovation to Sustainable Development Goal 3 and the lack of prediction software using AI in the diagnosis of children at risk for tuberculosis.

## RESUMEN

**Objetivos:**

desarrollar un prototipo de aplicación móvil utilizando Inteligencia Artificial (IA) para predecir y apoyar el diagnóstico de tuberculosis pulmonar en niños – TB *Kids.*

**Métodos:**

investigación de desarrollo tecnológico del tipo prototipado, basado en el modelo de *Rational Unified Process* y sus cuatro etapas: concepción, elaboración, construcción y transición. El desarrollo del prototipo de TB *Kids* se llevó a cabo desde noviembre de 2022 hasta julio de 2023.

**Resultados:**

el prototipo TB *Kids* cuenta con funcionalidades para evaluación de riesgos, evaluación nutricional, prueba cutânea de tuberculina, investigación de terapia antibiótica y contactos, interpretación de radiografías de tórax mediante IA con gráfico de riesgo y toma de decisiones, orientación adicional y registro del cuadro clínico.

**Conclusiones:**

el prototipo de aplicación móvil de alta fidelidad tiene una interfaz consistente, respondiendo con creatividad e innovación al Objetivo de Desarrollo Sostenible 3 y a la falta de software de predicción que utilice IA en el diagnóstico de niños en riesgo de tuberculosis.

## INTRODUCTION

By declaring the Sustainable Development Goals (SDGs) in 2015, the United Nations (UN) mobilized international agreements and a series of regional and national policies to transform the world through Agenda 30. The 17 goals cover different dimensions of life on the planet and, in particular, human life with greater justice and quality. The third goal is perhaps the one that most directly refers to health, “ensuring healthy lives and promoting well-being for all at all ages” which includes controlling diseases that overburden health systems and reproduce geopolitical disparities and health inequities. This goal is divided into nine targets, the third of which directly addresses tuberculosis (TB) (3.3 - End the epidemics of AIDS, TB, malaria and neglected tropical diseases, and combat hepatitis, waterborne diseases and other communicable diseases by 2030)^(1)^.

In order to monitor SDG indicators, the World Health Organization (WHO) developed a framework with 14 indicators that are associated with TB, recognizing that social, economic and health-related indicators interact in the incidence of this disease^(2)^. Investments in TB control measures are a result of significant advances in the disease and the difficulty in controlling it, a disease that could be prevented or cured. It is estimated that TB infection affects a quarter of the world’s population and that the disease progresses to approximately 10 million people diagnosed annually, with 1 million being children. Approximately 1.3 million deaths are associated with TB each year, making it one of the ten most serious causes of infant death^(3)^.

In 2022, the Ministry of Health (MoH) launched the nursing protocol for advanced practice of professionals in the face of TB, expanding access and all dimensions of care and qualified detection for the disease. The protocol intensifies and recognizes the expansion of advanced practice of nurses regarding comprehensive and assertive actions for TB, which are aimed at the early detection of active TB and latent tuberculosis infection (LTBI), optimization of the clinical management of confirmed cases, effectiveness of directly observed treatment and health surveillance of cases. Such actions require coordinated strategies and continuous monitoring by the Primary Health Care team^(4)^.

Nursing has been decisive in the control, screening and diagnosis of TB cases. The National Plan to End TB highlights that nurses have a decisive role in the development of strategies to eliminate the disease. Among the proposals highlighted, full participation of this professional in the implementation of new technologies for disease diagnosis and treatment, intensification of active search, qualification of actions to combat TB and better strengthening of control actions for vulnerable groups stood out^(4, 5)^.

In 2024, the theme of World TB Day was “Yes! We can end TB!”, in a manifesto to motivate the WHO’s goal of eliminating TB as a public health concern by 2035, which means facing the challenges that unfold in each country’s scenarios. Even considering results of stagnation or setbacks in the fight against TB impacted by the COVID-19 pandemic^(6, 7, 8)^, there are persistent challenges in response to TB related to specific population groups, such as indigenous people, children, people deprived of liberty and homeless people^(9, 10, 11)^, in addition to challenges related to screening, early diagnosis and preventive treatment^(12)^.

The current onverview of TB in Brazil and worldwide highlights the need for strong commitment and investment to achieve the SDG 3 targets by 2030, as the progress achieved is still far from complete success. This includes consistent action in terms of qualifying services and professionals, in addition to the use of new technologies.

The guidelines for coping with TB management highlight the use of chest radiography as a consolidated recommendation for detecting the disease. Radiography is a sensitive (combined sensitivity of 98%) and effective screening tool. Although it does not confirm TB diagnosis due to lack of specificity, it has a considerable role in detecting the disease in exposed children^(2)^.

In recent years, Artificial Intelligence (AI) and computer-aided detection software have been developed to augment and automate the interpretation of digital chest radiography in TB screening^(13)^. In a literature search for studies on AI focused on chest radiograph and TB analysis, 110 results were identified, of which 21 were published in the last five years^(13, 14, 15)^. It is important to note that only two of the 21 articles included studies with validated algorithms for children <15 years old, highlighting the need for clinical studies of AI in the pediatric imaging segment^(16, 17)^.

Many countries have sought to accelerate the adoption of mobile health applications, as demonstrated during the COVID-19 pandemic, when mobile technologies that enabled healthcare services represented alternatives to poor access to multidisciplinary healthcare for unassisted people in remote areas or far from hospital institutions^(18)^.

Control actions for pulmonary TB, in addition to prevention, are mainly focused on two essential actions such as early diagnosis of cases and timely disease treatment. Early recognition of TB symptoms in children is essential for carrying out appropriate tests and treatments. However, timely identification of TB in this age group has been a major challenge due to the complexity of diagnosis (symptoms and clinical signs that are often nonspecific). In this regard, it is important to improve and produce new technologies for early diagnosis of the disease, capable of enabling effective management and reducing the time between diagnosis and the start of treatment, since children are at greater risk of progression to severe forms^(19)^.

In view of this, the question is: what tools/functionalities and content should a mobile application contain to support the diagnosis of TB cases in children?

## OBJECTIVES

To develop a mobile application prototype using AI to predict and support the diagnosis of pulmonary TB in children.

## METHODS

### Ethical considerations

This project used a public image database and did not require submission to the ethics committee.

### Study design

This is a technological development research of the prototyping type using an AI system for interpreting chest radiographs together with a validated scoring diagnostic score, proposed by the MS^(4)^. The Rational Unified Process (RUP) was used, which is an interactive and flexible process methodology, innovative in relation to the linear model because it allows the accommodation of new requirements, changes in objectives or risk resolution throughout the process^(20)^.

### Study site and technical team

The development of the TB Kids prototype took place from November 2022 to July 2023 in Manaus, Amazonas, Brazil, together with the *Universidade do Estado do Amazonas* (UEA) Graduate Program in Public Health Nursing.

The technical team for producing the technology was composed of two nurses, who were the project’s creators, two scientific initiation scholarship holders, a software engineer, a designer and a nurse from the Municipal Department Tuberculosis Control Program Center. Weekly meetings were held, in person or via the Google Meet virtual platform, with the programming team in order to ensure alignment and continuous progress in the prototype development.

### Study stages

The RUP methodology has four phases (conception, elaboration, construction and transition), containing several interactions in each phase and with a complete development cycle, from requirements analysis to implementation, testing and final executable version.

The conception stage included: a) understanding the product, main focus and approach; b) structuring the content and functionalities as well as the approval of requirements and interfaces; c) defining the prototype design (iconography, typography and color palette) and creating the application logo.

The entire process was operationalized through the sprint planning meeting, which consisted of weekly meetings between researchers, IT staff and the healthcare service to develop the project content, taking into account the official guidelines and documents of MoH for TB control. In a non-linear manner, the literature search, meetings, visits to healthcare services and consultations of documents were carried out throughout the entire process, maintaining a continuous and updated approach. During the scoping review, the JBI methodology^(21)^ was used, which presented a structured model to identify and validate content and functionalities according to the scientific literature.

In the development stage, the application screens were created, foll owed by real-time testing to monitor errors, imp rove and reformulate. The goal was to eliminate the main risks and develop a stable architecture for the application, which involved creating a link between the front-end and back-end phases and training AI to read and interpret radiological images. Management used the scrum methodology, ensuring control over the entire process of creating the screens, from registration/login, navigation menu to using AI and creating the educational board.

Use case diagrams were used to describe functionalities and their interactions with users, sequences of actions and variants for a given actor, providing observable results, in order to express and document the behaviors of how the user interacts with the system^(20, 22)^.

In the construction stage, the prototype and its development and construction stages were presented, consisting of the TB Kids prototype and usability testing, carried out by the development team with the aim of identifying errors or adding information according to the assessments. For the usability tests, a checklist-type instrument proposed by Krone (2013) and usability testing involving Nielsen users (ISO9241-11 standard) were used^(23, 24)^.

This study did not include the transition stage (implementation of technology and product registration), which is essential for the dissemination and use of the application in clinical practice and public health.

## RESULTS

TB Kids refers to a high-fidelity prototype of a hybrid application, for Android and iOS platforms using AI, capable of assessing children with respiratory symptoms of TB and assuming the potential risk for the disease, using an AI system that identifies pulmonary changes suggestive of the disease and assesses the probability of a child having pulmonary TB by calculating a clinical score validated by the MoH^(4)^.

It is aimed at medical professionals and nurses who work in remote areas where other types of diagnostics are difficult to access. Moreover, it is a tool to support decision-making, conduct and guidance related to childhood TB based on evidence, validated risk classification score and real-time reading of chest X-rays.

To understand the product, main focus and approach, a survey was carried out of shapes, colors and typography used in various visual elements, as well as distribution, arrangement and layout of elements in interfaces of systems and applications present in the global market.

The relevant content identified in the scoping review stage, as well as the functionalities, were detailed. The requirements and interfaces were approved with the aim of providing a pleasant and intuitive user experience. Chart 1 presents the functionalities and interfaces included.

**Chart 1:** Description of screens, contents and functionalities of the TB Kids prototype

TB Kids prototype		
SCREENS	CONTENT	FUNCTIONALITY
**Professional registration/login**	Professional name, place of work, telephone number and password.	Professional login for software access and availability.
**Risk score screens**		
**Score screen 1** **Nutritional assessment**	Fill in data regarding weight, height, age and sex. The calculation will be generated through the application based on the growth interpretation chart, and will present the following results: thinness, severe thinness, eutrophy, risk of overweight, overweight, obesity. - Severe malnutrition (weight < 10th percentile) corresponds to 5 points. - Weight ≥ 10th percentile corresponds to 0 points.	Investigate the risk signaling for malnutrition according to the information. - Weights between the 10^th^ and 3^rd^ percentiles characterize a risk situation or nutritional alert. - Weights between the 3^rd^ and 0.1 percentile represent low weight for age (or insufficient weight gain). - Values below the 0.1 percentile represent very low weight for age.
**Score screen 2** **Child symptoms screen**	- Child has had a fever or symptoms such as cough, weakness, expectoration, weight loss, sweating for two weeks or more. Yes answer: 15 points. - Asymptomatic or with symptoms for less than two weeks. Yes answer: 0 points.	Investigate symptoms for active TB.
**Score screen 3** **Antibiotic therapy performed**	- Did the child have a respiratory infection that improved after using antibiotics for common germs or without antibiotics? Decrease 15 points.	Assess whether children improved after using common antibiotics; if not, there was presumed TB.
**Score screen 4** **Instigation of contact of adults with TB**	Investigating whether the child was a close contact in the last two years of an index case for active TB corresponds to 10 points. Occasional or negative corresponds to 0 points.	Record and investigate risk factors with a strong predisposition to TB.
**Score screen 5** **Tuberculin test analysis**	Did the child undergo PPD? PT≥10 mm/10 points. PT between 5 and 9 mm/5 points. PT 5 ≤ mm/0 points. Absence of the above criteria.	Record and investigate risk factors with a strong predisposition to TB.
**Score screen 6** **Radiological picture analysis**	- Capture the radiograph image for identification by AI: presence of hilar adenomegaly or miliary pattern and/or condensation or infiltrate (with or without excavation). Time: unchanged for ≥ two weeks. - Presence of condensation or infiltrate (with or without excavation)? Time: ≥ two weeks. Yes answer: 15 points. - Condensation or infiltrate of any type for less than two weeks. Yes answer: 5 points. - Normal radiograph. Decrease 5 points.	Perform a real-time reading of the chest X-ray of children being investigated, using the application, using the AI system. AI will analyze the radiological changes in the images; then, the score will be filled in automatically, generating a score and decision-making.
**Score screen 7** **Result with presentation of a graph with the measurement of risk and indication of conduct**	Generation of a risk graph presenting the results and analysis of each scored item with the measurement of risk and indication of conduct according to the score. Presentation of results through the risk graph and exposure of children to TB.	Presentation of results through a graph of the child’s risk and exposure to TB according to score analysis. High risk for TB: >40; moderate risk: between 30 and 35; and low risk: >25. Direction based on the result. High score - start treatment. The basic regimen in children (<10 years of age) consists of three drugs, in the intensive phase (RHZ), and two drugs, in the maintenance phase (RH), with individualized pharmacological presentations (tablets and/or suspension). Moderate score: guidance to start treatment at the physician’s discretion. Low score: guidance on other examination options for investigation.
**Screens of children’s clinical picture**		
**Screen showing children’s clinical and radiological results**	Risk chart, score result, child assessment history, nutritional data, symptoms and image storage.	Viewing the record and storage of each child’s assessment for professional monitoring and outcome.
**Complementary guidance screens**		
**Additional guidance screen**	Basic treatment scheme.	A table with drug schemes and flowcharts will be made available to guide each treatment approach.
**Complementary diagnostic method screen**	gastric lavage, PCR, bronchoalveolar lavage, AFB, swab.	Guidance on the execution and direction of examinations that assist in decision-making.
**Adverse events screen**	Possible adverse events that occur in children during treatment.	Assist professionals with adverse events that occur during the use of medication.
**Conduct flowchart screen**	Flowchart of conduct for investigation and assessment of active TB and LTBI.	Directs professionals according to the criteria for active TB or LTBI.
**Ministry of Health Manuals**	TB Recommendation Manuals.	Professionals will have access to the most up-to-date procedures.

*Source: adapted from Sant’ Anna et al. (2006) and Ministry of Health (2019).*

To assess the risk of TB, the instrument recommended by the Ministry of Health for risk classification scoring to support the diagnosis of pulmonary TB in children and adolescents was used, divided into four items: clinical and radiological findings; contact with an adult with TB; tuberculin skin test; and nutritional status. Each response given to these items is converted into a specific score^(4)^.

For the assessment interface of children’s Body Mass Index (BMI), the percentile chart used by the MoH was used, employing children’s weight, height, sex and age measurements, obtained in the physical examination, transformed into algorithms and translated into the software language^(25)^.

According to the prototype designer, the chosen icons are visual representations of key concepts related to the application, such as child, x-ray, lung, drops and graphs. These icons were selected to directly communicate the purpose of the application, which is to assist in the early identification and diagnosis of TB in children, using prediction technology and clinical support.

In the elaboration stage, an information flow was created to direct and define the actors who will interact with the TB Kids systems and functions, in order to align real-world programming with the computational language using a use case diagram and its user interface requirements.

Using scrum for project management, mockups were created to organize and understand the application dynamics. Therefore, this stage brings together the front-end, back-end, AI training and production of screens with their interfaces.

In the front-end development, the designer proposed concatenation and its adaptation with the functions intended for users’ response, through calculations and abstract representations of variables that are later sent to the server and database, as well as visual style sheets that define the graphic guidelines in lists of definitions that combine values, measurements, sizes, colors and other aesthetic parameters. At this stage, the user experience was analyzed through internal tests on different types of cell phones.

In the back-end development, the tables and methods of communication between the user and the server were defined, optimizing the use of data as much as possible and organizing the information to be accessed in queries by users registered in the system and in analyses performed with potential patients. The unified modeling language diagram outlined the software dynamics and flow, describing its functionalities and content.

AI-based image training followed the criteria for filtering, editing, and using chest X-ray images to categorize and feed the AI neural training platform. In parallel, a dynamic and local (offline) solution was generated to use the results obtained by AI, through the export of use-oriented libraries in Javascript language, designed to be easily updated and integrated into web browsers and applications. The standard public digital image database for TB created by the National Library of Medicine in collaboration with the Department of Health and Human Services, Montgomery County, Maryland, USA, available at https://openi.nlm.nih.gov/faq#collection/https://nihcc.app.box.com/v/ChestXray-NIHCC. The sample for this first stage included 799 chest radiographs treated and categorized as normal and abnormal with manifestations of TB, 138 from the Montgomery (USA) image database (80 normal chest radiographs/without lung changes and 58 cases of radiological images with manifestations of TB) and 662 from the Chinese image database (326 cases of normal radiographs/without lung changes and 336 cases indicating radiological changes for TB)^(26)^.

The Teachable Machine platform was chosen to train the AI of the TB Kids prototype, integrated with the rest of the system by importing the trained model in TM format, using TensorFlow technology for JavaScript. TensorFlow is an open-source library for large-scale numerical computation and machine learning. TensorFlow was responsible for grouping a series of machine learning and deep learning (neural networks) models and algorithms. All algorithms related to image prediction and machine learning are related to Tensorflow and the Teachable Machine platform^(27)^.

**Figure 1:**
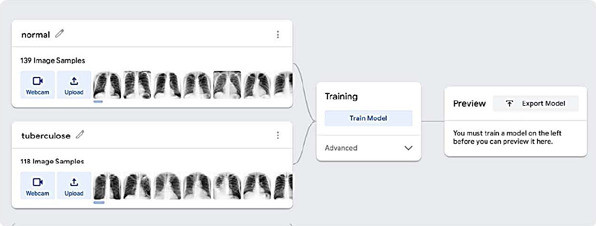
Categorizing images for Artificial Intelligence training

The initial stage for training to begin was to standardize the collected images, eliminating all textual residue from the images and formatting them in a 1:1 (one to one) ratio of 224px/224px. The second stage was to create categories on the training platform so that AI could, through its neural networks, learn to differentiate healthy lungs from lungs with TB (Figure 1). The third stage was training, exporting the model and implementing the dynamic connection on the Teachable Machine platform.

Once AI was trained, the system needed to be integrated with graphical interfaces. The fourth stage was to adapt the model to a data structure that would allow validation and testing of results. Using the VSCode platform and the Javascript, CSS, PHP and HTML languages, a web application was created that could use the model and analyze the results based on comparison with previous machine learning. All radiographs were properly divided, processed and categorized before being used in training.

The construction stage consists of presenting the TB Kids prototype, which can be accessed via a link. To access it, one must register and create a password, which will be stored in encrypted form in the database. Internally, the applications share the same database, thus enabling interoperability and data integrity.

**Figure 2:**
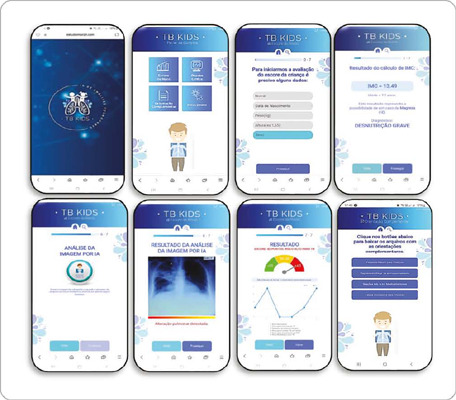
TB Kids prototype screens

The TB Kids application’s Splash Screen provides information about the application, such as the software’s objective, target population, and how it will be used, welcoming the professionals who will access it (Figure 2).

In Figure 2, on the second screen (control panel), users will have access to the four main features of the application. After navigating through screens 1 to 5 of the risk score, answering specific questions, professionals will have the option of attaching the image or pointing the camera at the x-ray that they would like to read. The application will start the analysis and generate a diagnostic result of the image and, if it identifies abnormalities, it will indicate the lung alteration detected as a result.

It also shows the result screen of the score generated according to children’s risk level for TB. The interpretation follows the MoH manual: score above or equal to ≥ 40 points: very likely diagnosis (start treatment); 30 to 35 points: possible diagnosis (start treatment at the doctor’s discretion); ≤ 25 points: unlikely diagnosis (investigate and use other methods)^(4)^.

TB Kids also offers the option of additional guidance on possible approaches based on the result: high score - start treatment and professionals will have access to the basic treatment regimen for children according to their weight and age (< 10 years of age), consisting of three drugs in the intensive phase (RHZ) and two in the maintenance phase (RH), with individualized pharmacological presentations (tablets and/or suspension); low score - will be guided to other options for investigation tests (gastric lavage, PCR, bronchoalveolar lavage, BAAR, swab), and each test will have videos guiding the professionals in its execution. Guidance on possible adverse events during treatment and LTBI diagnostic algorithms are also available, understanding the importance of a comprehensive and effective assessment for TB in this age group.

Assessment resulted in seven violated heuristics out of the ten proposed by Nielsen^(24)^, and 38 problems were identified in the 22 screens of the TB kids prototype described in Table 1.

**Table 1:** Violated heuristics, usability issues and severities found in prototype screens, Manaus, Amazonas, Brazil, 2023

Heuristic violated	Usability issues (percentage)	Severities					Total
		0	1	2	3	4	
H1- Visibility of system state	7 (18.42%)			7			7
H2- Correspondence between the system interface and the real world	2 (5.26%)					2	2
H3- User control and freedom	7 (18.42%)				1		1
H4- Consistency and standard	10 (26.32%)		1				1
H5- Error prevention	2 (5.26%)		1	1			2
H7- Efficiency and flexibility of use	2 (5.26%)		2				2
H8- Aesthetics and minimalist design	8 (21.05%)		8				8
Total by heuristics	38 (100%)		12	8	1	2	38

The results showed that “consistency and standard” and “minimalist aesthetics and design” were the most violated heuristics, with ten (26.32%) and eight (21.05%) usability problems, followed by “visibility of system state” and “user control and freedom”, with seven (18.42%) items violated in each heuristic assessed.

The prototype was tested by four evaluators, and their main comments using heuristics were: need for design improvement, with changes to the prototype’s color palette; the screens were cluttered and dark, not following standards; missing date of birth mask; need to insert forward and back buttons on the screens, allowing users to control their actions; there was also a lack of stages marked on the risk score screens and fields filled in on the buttons on the PPD assessment screens, needing to be unmarked by professionals.

The usability problem that received the highest severity rating^(4)^ was the heuristic “correspondence between the system interface and the real world”, indicating that the prototype did not generate the correct score in the risk score and, consequently, interfered with the diagnostic result. Another item assessed in the same level was inaccuracy in generating results on the assessment screen for calculating children’s BMI; the error was immediately corrected in both items, and retests were performed by evaluators.

As for prototype corrections, changes were made to the design, with changes to the prototype colors, making the technology more intuitive and with less content pollution, and organizing the content by topic in an easy-to-understand language. Explanations about the graph were added, and the prototype had usage information on the home screen. After the bug was fixed, the correct score was obtained for each item assessed, generating a compatible result. The forward and back buttons were also added, marked and centered on each screen. In relation to the BMI calculation, there was an adjustment according to the percentile calculation according to the MoH, which was updated and tested, and for the PPD assessment screen, pre-marked fields were removed, and the fields for filling in were adjusted.

## DISCUSSION

Without treatment, the TB mortality rate is high (50%), while the cure rate with the treatments recommended by the WHO is around 85%, which demonstrates the importance of access to early diagnosis, combined with multisectoral actions on determining factors of TB, such as poverty, malnutrition, HIV infection, smoking and diabetes^(2)^. Healthcare providers’ knowledge about screening and detection of childhood TB cases is crucial for the best attitudes and practices, and the effectiveness of contact investigation in health units and the community, which is related to the availability of resources and the provision of specific training^(9)^.

The leading role of nursing in tackling TB and the importance of technically based information to expand the potential of experiences in the daily routine of services are recognized, especially when these tools strengthen professionals’ autonomy in their work, are easy to apply and are produced in a participatory manner^(27)^. With proper training, TB Kids can become a valuable tool to support the diagnosis of these professionals in the fight against the disease in children in the Amazon region and other areas affected by TB. Among the studies developed using images to feed AI, it is still challenging to have an image database of chest X-rays of children diagnosed with TB^(15, 16)^.

The update of Resolution 736 of January 17, 2024 by the Federal Nursing Council regarding the nursing process provides for nurses’ autonomy in assessment through imaging tests, being an important support for nursing conduct, outcomes and diagnoses for this professional^(28)^.

TB Kids technology can be a crucial tool to support nurses’ interpretation and clinical judgment, as it is aimed at orderly care and more assertive decision-making for a patient at risk for TB. By offering sensitive support in identifying significant radiological changes for the disease, it provides support for diagnosis in a systematic and careful manner, allowing the measurement (scores) of the chances of a child being affected by TB, proposing the best possible therapeutic approach for this population and reducing the potential risks of aggravation and late diagnosis.

The educational resources and support materials added to TB Kids aim to strengthen nurses’ and healthcare professionals’ knowledge and training in tackling TB in children. By providing this information, the application seeks to improve quality of care and reduce the weaknesses faced by professionals in caring for children with a presumptive diagnosis of TB.

Another advantage of this technology is that nurses can also be guided towards diagnostic investigation procedures for the latent form of the disease. Professionals will be guided towards algorithms and investigation flowcharts for both active TB and LTBI, highlighting the importance of comprehensive technologies that address all forms of the disease, since detecting the latent form is a decisive factor in interrupting the chain of transmission and eliminating TB^(29)^.

By providing an advanced technological tool to support TB diagnosis in children, TB Kids can contribute to the elimination of TB and directly contribute to achieving the SDG targets. Eliminating TB requires a renewed commitment to equity, social justice and the use of innovative technologies. Integrating these elements into public health policies and specific programs, such as TB Kids, cannot only accelerate progress towards SDG 3, but also contribute to sustainable and inclusive development.

It is important to consider, when developing mobile applications to support diagnostics, the recent changes in the General Data Protection Law (GDPL), with the establishment of clear rules on the processing of personal data and standardization of standards. As for children, GDPL states that specific consent from those responsible for processing a child’s personal data is required^(30)^.

It is worth noting that the training approach with a diverse and representative set of images is crucial to ensure accuracy and reliability of results obtained by TB Kids. The quality and quantity of images are determining factors for the AI system performance.

Furthermore, the usability test carried out provides a level of expertise intrinsic to the process, ensuring an in-depth and specialized analysis of the system. The use of a structured checklist and Nielsen severity classification offers a systematic and rigorous methodology for identifying, recording and prioritizing failures, providing a solid basis for implementing substantial improvements in the system being assessed^(24)^.

Using tools like TB Kids directly aligns with UN SDG 3, which aims to ensure healthy lives and promote well-being for all at all ages. TB Kids not only supports healthcare professionals in diagnosing and treating childhood TB, but also contributes to reducing health disparities and strengthening health systems.

### Study limitations

One limitation of this study was the fact that the transition stage was not considered. In the future, we intended to train users and register the product with the UEA Innovation and Technology Agency for later availability in online stores. Therefore, for the stages of assessing the application reliability and performance, we expect to collect prospective X-rays of children in reference units in the State.

### Contributions to nursing, health or public policy

The prototype developed is a creative and innovative tool that responds to the current lack of available tools that use AI to diagnose children at potential risk for TB, thus representing an important contribution to tackling childhood pulmonary TB in Primary Health Care. This technology brings benefits, such as a considerable reduction in diagnosis time and increased accuracy of clinical assessments, facilitating the early identification of TB cases in children. Furthermore, by integrating AI functionalities, the prototype supports nursing professionals in identifying and continuously monitoring patients, contributing to adequate follow-up and effective management of the disease. In this way, it strengthens nursing practices and improves the quality of care provided in Primary Health Care.

## CONCLUSIONS

The TB Kids prototype is a transformative proposal for nursing work in controlling childhood TB, since technologies that use AI to support TB diagnosis for this population are limited and the broader scope of nursing work in this field needs to be consolidated. In addition to the assessment for active TB, it has the differential of being able to investigate the latent form of the disease as a complete and dynamic tool that offers professionals an application for continuity and quality of care. Another advance that technology brings is that it offers professionals access to educational materials for training and improving knowledge, knowing that technologies with educational support are fundamental devices for quality of care, in addition to being a privileged focus of nursing intervention.

It is concluded that the TB Kids prototype represents a significant advance in the fight against childhood pulmonary TB. The scarcity of digital tools to support diagnosis for children makes TB Kids an innovative and empowering tool in the context of public health. However, validating intelligent tools for use in real-world scenarios is challenging, since the availability of accurate image databases for this age group remains a limitation. Therefore, further studies are needed, and the contributions of this technological product are not intended to replace human experts.
