# Supplementary material for: Mobile application prototyping using Artificial Intelligence to support childhood tuberculosis diagnosis
Source: Rev Bras Enferm. 2025 Sep 8;78(3):e20240398. doi: 10.1590/0034-7167-2024-0398 (PMC12419783; doi:10.1590/0034-7167-2024-0398)
Supplement: Translation

# Translation: Prototipagem de aplicativo móvel utilizando Inteligência Artificial para apoio ao diagnóstico da tuberculose infantil

**DOI:** 10.1590/0034-7167-2024-0398pt

**Authors:** Katerine Souza Picoli, Flávia Regina Souza Ramos, Denise Maria Guerreiro Vieira da Silva, Bruno da Veiga Thurner, Daniel Souza Sacramento, Irineide Assumpção Antunes, Lucas Lorran Costa de Andrade, Amélia Nunes Sicsú

**Affiliations:** I Universidade do Estado do Amazonas. Manaus, Amazonas, Brasil; II Universidade Federal de Santa Catarina. Florianópolis, Santa Catarina, Brasil; III Universidade Federal de Santa Maria. Santa Maria, Rio Grande do Sul, Brasil; IV Secretaria Municipal de Saúde de Manaus. Manaus, Amazonas, Brasil; V Secretaria do Estado da Saúde do Amazonas. Manaus, Amazonas, Brasil

**Keywords:** Aplicativos Móveis, Inteligência Artificial, Tuberculose Pulmonar, Criança, Diagnóstico

## Abstract

**Objetivos:**

desenvolver protótipo de aplicativo móvel utilizando Inteligência Artificial (IA) para a predição e apoio ao diagnóstico da tuberculose pulmonar em crianças – *TB Kids.*

**Métodos:**

pesquisa de desenvolvimento tecnológico do tipo prototipagem, baseada no modelo *Rational Unified Process* e suas quatro etapas: concepção, elaboração, construção e transição. O desenvolvimento do protótipo *TB Kids* ocorreu no período de novembro de 2022 a julho de 2023.

**Resultados:**

o protótipo *TB Kids* possui funcionalidades de avaliação de risco, avaliação nutricional, prova tuberculínica, investigação de antibioticoterapia e de contatos, interpretação de radiografias de tórax através da IA com gráfico de risco e tomada de decisão, de orientações complementares e registro do quadro clínico.

**Conclusões:**

o protótipo de aplicativo móvel de alta fidelidade é de consistente interface, respondendo com criatividade e inovação ao Objetivo de Desenvolvimento Sustentável 3 e à carência de *softwares* de predição utilizando IA no diagnóstico de crianças com risco para tuberculose.

## INTRODUÇÃO

Ao declararem os Objetivos de Desenvolvimento Sustentável (ODS), em 2015, a Organização das Nações Unidas (ONU) mobilizou acordos internacionais e uma série de políticas regionais e nacionais para transformar o mundo por meio da Agenda 30. Os 17 objetivos recobrem diferentes dimensões da vida no planeta e, em especial, a vida humana com mais justiça e qualidade. O terceiro objetivo talvez seja aquele que de forma mais direta remete à saúde, “Assegurar uma vida saudável e promover o bem-estar para todos, em todas as idades”, que inclui o controle de doenças que sobrecarregam os sistemas de saúde e reproduzem disparidades geopolíticas e inequidades em saúde. Este objetivo se divide em nove metas, sendo a terceira diretamente dirigida à tuberculose (TB) (3.3 - Até 2030, acabar com a epidemia de AIDS, TB, malária e doenças tropicais negligenciadas, além de combater a hepatite, doenças transmitidas pela água e outras doenças transmissíveis)^(1)^.

Com a finalidade de monitorar os indicadores dos ODS, a Organização Mundial da Saúde (OMS) desenvolveu um quadro com 14 indicadores que estão associados à TB, reconhecendo que indicadores sociais, econômicos e relacionados à saúde interatuam na incidência dessa doença^(2)^. Os investimentos em ações para o controle da TB decorrem de seus expressivos avanços e dificuldade de controle de uma doença que poderia ser prevenida ou curada. Estima-se que a infecção por TB atinja um quarto da população mundial e a evolução para a doença se aproxime de 10 milhões de pessoas diagnosticadas anualmente, com 1 milhão em crianças. Cerca de 1,3 milhões de mortes são associadas à TB por ano, estando entre as dez causas mais graves de morte infantil^(3)^.

O Ministério da Saúde (MS) lançou, em 2022, o protocolo de enfermagem para a prática avançada do profissional diante da TB, ampliando o acesso e todas as dimensões do cuidado e detecção qualificada para a doença. O protocolo intensifica e reconhece a ampliação da prática avançada do enfermeiro quanto às ações integrais e assertivas para TB, sendo elas direcionadas à detecção precoce da TB ativa e infecção latente da tuberculose (ILTB), otimização do manejo clínico dos casos confirmados, efetividade do tratamento diretamente observado e vigilância em saúde dos casos. Tais ações necessitam de estratégias articuladas e monitoramento contínuo pela equipe da Atenção Primária à Saúde^(4)^.

A enfermagem tem sido decisiva no controle, rastreio e diagnóstico de casos de TB. O Plano Nacional pelo Fim da TB destaca que o enfermeiro tem um protagonismo determinante na construção de estratégias de eliminação da doença. Das propostas pontuadas, destacaram-se a participação integral desse profissional na implementação de novas tecnologias para o diagnóstico e tratamento da doença, intensificação da busca ativa, qualificação de ações de enfrentamento para TB e um melhor fortalecimento de ações de controle para os grupos vulneráveis^(4, 5)^.

Em 2024, o tema do Dia Mundial da TB foi “Sim! Nós podemos acabar com a TB!” em um manifesto para motivar a meta da OMS de eliminar a TB como problema de saúde pública até 2035, o que significa enfrentar os desafios que se desdobram nos cenários de cada país. Mesmo considerando resultados de estagnação ou retrocesso na luta contra a TB impactados pela pandemia de COVID-19^(6, 7, 8)^, existem desafios persistentes na resposta à TB relacionados a grupos populacionais específicos, como indígenas, crianças, pessoas privadas de liberdade e moradores de rua^(9, 10, 11)^, além dos desafios relacionados ao rastreio, diagnóstico precoce e tratamento preventivo^(12)^.

O panorama atual da TB no Brasil e no mundo alerta sobre a exigência de forte empenho e investimento para o alcance das metas do ODS 3 até 2030, pois os avanços alcançados ainda estão longe do pleno sucesso. Isso inclui consistente atuação em termos de qualificação de serviços e profissionais, além do uso de novas tecnologias.

As diretrizes de enfrentamento para o manejo da TB destacam o uso da radiografia de tórax como recomendação consolidada para detecção da doença. A radiografia é um instrumento de triagem sensível (sensibilidade combinada de 98%) e efetiva. Embora não defina a confirmação do diagnóstico de TB por carência de especificidade, ela possui uma função considerável na detecção da doença em crianças expostas^(2)^.

Nos últimos anos, a Inteligência Artificial (IA) e *software* de detecção assistida por computador foram desenvolvidos para aumentar e automatizar a interpretação da radiografia digital de tórax na triagem de TB^(13)^. Em uma busca na literatura por estudos sobre IA voltados para análises de radiografias torácicas e TB, foram identificados 110 resultados, dos quais 21 foram publicados nos últimos cinco anos^(13, 14, 15)^. É importante salientar que somente dois dos 21 artigos incluíram estudos com algoritmos validados para crianças <15 anos, evidenciando a necessidade de estudos clínicos de IA no segmento de imagem pediátrica^(16, 17)^.

Muitos países têm buscado acelerar a adoção de aplicativos móveis em saúde, como foi demonstrado durante a pandemia de COVID-19, quando tecnologias móveis que viabilizassem serviços de saúde representavam alternativas diante do deficiente acesso à assistência multiprofissional à saúde por pessoas desassistidas em áreas remotas ou longe de instituições hospitalares^(18)^.

As ações de controle da TB pulmonar, para além de sua prevenção, são focadas principalmente em duas ações essenciais: o diagnóstico precoce dos casos e o tratamento oportuno da doença. O reconhecimento precoce dos sintomas de TB em crianças é primordial para a realização de testes e tratamentos adequados. Todavia, a identificação em tempo hábil da TB nessa faixa etária tem sido um grande desafio, devido à complexidade do diagnóstico (sintomas e sinais clínicos muitas vezes inespecíficos). Nesse sentido, é importante aperfeiçoar e produzir novas tecnologias de diagnóstico precoce da doença capazes de viabilizar manejo efetivo e redução do tempo entre o diagnóstico e início do tratamento, já que crianças correm um risco maior de progressão para as formas graves^(19)^.

Diante disso, questiona-se: quais as ferramentas/funcionalidades e conteúdos devem conter aplicativo móvel para o apoio ao diagnóstico de casos de TB em crianças?

## OBJETIVOS

Desenvolver protótipo de aplicativo móvel utilizando IA para a predição e apoio ao diagnóstico da TB pulmonar em crianças.

## MÉTODOS

### Considerações éticas

Este projeto utilizou banco de imagens público, não necessitando de submissão ao comitê de ética.

### Tipo de estudo

Trata-se de pesquisa de desenvolvimento tecnológico do tipo prototipagem, utilizando um sistema de IA para interpretação de radiografias torácicas juntamente com um escore de diagnóstico de pontuação validado proposto pelo MS^(4)^. Foi utilizado o *Rational Unified Process* (RUP), que é uma metodologia de processo interativo e flexível, inovador em relação ao modelo linear, por permitir a acomodação de novos requisitos, mudanças de objetivos ou solução de riscos ao longo do processo^(20)^.

### Local e equipe técnica do estudo

O desenvolvimento do protótipo *TB Kids* ocorreu no período de novembro de 2022 a julho de 2023 em Manaus, Amazonas, Brasil, junto ao Programa de Pós-Graduação em Enfermagem em Saúde Pública da Universidade do Estado do Amazonas (UEA).

A equipe técnica da produção da tecnologia foi composta por duas enfermeiras, que foram idealizadoras do projeto, dois bolsistas de iniciação científica, um engenheiro de *software,* um *designer* e um enfermeiro do Núcleo do Programa de Controle de Tuberculose da Secretaria Municipal. Foram realizadas reuniões semanais, presenciais ou por via plataforma virtual *Google Meet* com a equipe de programação, a fim de garantir o alinhamento e o progresso contínuo do desenvolvimento do protótipo.

### Etapas do estudo

A metodologia RUP possui quatro fases (concepção, elaboração, construção e transição), contendo várias interações em cada fase e com um ciclo completo de desenvolvimento, desde a análise de requisitos até implementação, testes e versão final executável.

A etapa de concepção incluiu: a) compreensão do produto, foco principal e abordagem; b) estruturação dos conteúdos e funcionalidades, assim como a aprovação dos requisitos e interfaces; c) definição do *design* do protótipo (iconografia, tipografia e paleta de cores) e criação da logomarca do aplicativo.

Todo o processo foi operacionalizado por meio do *sprint planning meeting,* que consistiu em reuniões semanais entre pesquisadores, equipe de TI e do serviço de saúde para construção do conteúdo do projeto, levando em consideração as diretrizes e documentos oficiais do MS para o controle da TB. De forma não linear, a busca na literatura, as reuniões, as visitas aos serviços de saúde e as consultas aos documentos foram realizadas ao longo de todo o processo, mantendo-se uma abordagem contínua e atualizada. Durante a revisão de escopo, foi utilizada a metodologia do JBI^(21)^, que apresentou um modelo estruturado para identificar e validar conteúdos e funcionalidades de acordo com a literatura científica.

Na etapa de elaboração, houve a criação das telas do aplicativo, seguida de testes em tempo real para acompanhamento de erros, aperfeiçoamento e reformulação. O objetivo foi eliminar os principais riscos e desenvolver uma arquitetura estável para o aplicativo, o que envolveu a produção do *link* entre as fases *front-end* e *back-end* e o treinamento da IA para leitura e interpretação de imagens radiológicas. O gerenciamento utilizou a metodologia *scrum,* garantindo o controle de todo o processo de criação das telas, do cadastro/*login,* menu de navegação até a utilização da IA e criação do quadro educativo.

Diagramas de casos de usos foram usados para descrever as funcionalidades e suas interações com os usuários, as sequências de ações e variantes para um determinado ator, fornecendo resultados observáveis, de modo a expressar e documentar os comportamentos de como o usuário interage com o sistema^(20, 22)^.

Na etapa de construção, foi realizada a apresentação do protótipo e suas etapas de desenvolvimento e construção, que consiste no protótipo *TB Kids* e teste de usabilidade, realizado pela equipe de desenvolvimento com o objetivo de identificar erros ou acrescentar informações de acordo com as avaliações. Para os testes de usabilidade, foram utilizados um instrumento tipo *checklist* proposto Krone (2013) e teste de usabilidade envolvendo usuários de Nielsen (Norma ISO9 241-11)^(23, 24)^.

Neste estudo, não foi contemplada a etapa de transição (implementação da tecnologia e registro do produto), fundamental para a disseminação e utilização do aplicativo na prática clínica e na saúde pública.

## RESULTADOS

O *TB Kids* refere-se a um protótipo de alta fidelidade de aplicativo tipo híbrido para as plataformas *Android* e *iOS* utilizando IA, capaz de avaliar crianças com sintomas respiratórios de TB e presumir o risco potencial para a doença, utilizando um sistema de IA que identifica alterações pulmonares sugestivas para a doença e avalia a probabilidade de uma criança ter TB pulmonar por meio do cálculo de escore clínico validado pelo MS^(4)^.

É direcionado a profissionais médicos e enfermeiros que atuam em áreas remotas e de difícil acesso a outros tipos de diagnósticos. Além disso, é uma ferramenta de apoio na tomada de decisão, condutas e orientações relacionadas à TB infantil baseadas em evidências, escore de classificação de risco validado e leitura em tempo real de radiografias de tórax.

Para compreensão do produto, foco principal e abordagem, foi realizado levantamento de formas, cores e tipografias utilizadas em elementos visuais diversos, bem como distribuição, arranjo e diagramação de elementos em interfaces de sistemas e aplicativos presentes no mercado mundial.

Foram detalhados os conteúdos relevantes identificados na etapa de revisão do escopo, bem como as funcionalidades. Os requisitos e as interfaces foram aprovados com o objetivo de proporcionar uma experiência de usuário agradável e intuitiva. As funcionalidades e interfaces incluídas estão apresentadas no Quadro 1.

**Quadro 1:** Descrição das telas, conteúdos e funcionalidades do protótipo *TB Kids*

Protótipo *TB Kids*		
TELAS	CONTEÚDO	FUNCIONALIDADE
**Cadastro do profissional/*login***	Nome do profissional, local que atua, número de telefone e senha.	Login do profissional para acesso e disponibilidade do *software.*
**Telas do escore de risco**		
**Tela 1 do escore** **Avaliação nutricional**	Preencher dados referentes ao peso, altura, idade e sexo. O cálculo será gerado através do aplicativo com base no gráfico de interpretação do crescimento, e apresentará os seguintes resultados: magreza, magreza acentuada, eutrofia, risco de sobrepeso, sobrepeso, obesidade. - Desnutrição grave (peso < percentil 10) corresponde a 5 pontos. - Peso ≥ percentil 10 corresponde a 0 pontos.	Investigar a sinalização de risco para desnutrição conforme informações. - Pesos entre os percentis 10 e 3 caracterizam uma situação de risco ou de alerta nutricional. - Pesos entre o percentil 3 e o percentil 0,1 representam peso baixo para a idade (ou ganho insuficiente de peso). - Valores abaixo do percentil 0,1 representam peso muito baixo para a idade.
**Tela 2 do escore** **Tela de sintomas da criança**	- Criança teve febre ou sintomas como tosse, adinamia, expectoração, emagrecimento, sudorese por duas semanas ou mais. Resposta sim: 15 pontos. - Assintomático ou com sintomas há menos de duas semanas. Resposta sim: 0 pontos.	Investigar os sintomas para TB ativa.
**Tela 3 do escore** **Realização de antibioticoterapia**	- Criança teve infecção respiratória com melhora após uso de antibióticos para germes comuns ou sem antibióticos? Diminuir 15 pontos.	Avaliar para esse quadro se a criança melhorou após o uso de antibióticos comuns; caso não, houve TB presumida.
**Tela 4 do escore** **Instigação do contato de adultos com TB**	Investigar se a criança foi contato próximo nos últimos dois anos de caso índice para TB ativa corresponde a 10 pontos. Ocasional ou negativo corresponde a 0 ponto.	Registrar e investigar fatores de risco com forte predisposição para TB.
**Tela 5 do escore** **Análise da prova tuberculínica**	Criança realizou o PPD? PT≥10 mm/10 pontos. PT entre 5 e 9 mm/5 pontos. PT 5 ≤ mm/0 pontos. Ausência dos critérios acima.	Registrar e investigar fatores com forte predisposição para TB.
**Tela 6 do escore** **Análise do quadro radiológico**	- Capturar a imagem da radiografia para identificação através da IA: presença de adenomegalia hilar ou padrão miliar e/ou condensação ou infiltrado (com ou sem escavação). Tempo: inalterado por ≥ duas semanas. - Presença de condensação ou infiltrado (com ou sem escavação)? Tempo: ≥ duas semanas. Resposta sim: 15 pontos. - Condensação ou infiltrado de qualquer tipo por menos de duas semanas. Resposta sim: 5 pontos. - Radiografia normal. Diminuir 5 pontos.	Realizar a leitura da radiografia de tórax, em tempo real, da criança investigada, pelo aplicativo, através do sistema de IA. Serão analisadas por IA as alterações radiológicas das imagens; logo, o escore será preenchido automaticamente, gerando uma pontuação e tomada de decisão.
**Tela 7 do escore** **Resultado com a apresentação do gráfico com a mensuração do risco e indicação da conduta**	Geração de gráfico de risco apresentando os resultados e análise de cada item pontuado com a mensuração do risco e indicação da conduta de acordo com a pontuação. Apresentação dos resultados através do gráfico de risco e exposição da criança para TB.	Apresentação dos resultados através do gráfico de risco e exposição da criança para TB de acordo com análise de pontuação. Risco alto para TB: >40; risco moderado: entre 30 e 35; e risco baixo: > 25. Direcionamento conforme o resultado. Escore alto - iniciar o tratamento. O esquema básico em crianças (< de 10 anos de idade) é composto por três fármacos, na fase intensiva (RHZ), e dois fármacos, na fase de manutenção (RH), com apresentações farmacológicas individualizadas (comprimidos e/ou suspensão). Escore moderado: orientação iniciar tratamento a critério médico. Escore baixo: será orientado a outras opções de exames para investigação.
**Telas do quadro clínico da criança**		
**Tela do resultado do quadro clínico e radiológico da criança**	Gráfico de risco, resultado do escore, histórico da avaliação da criança, dados nutricionais, sintomas e armazenamento de imagens.	Visualização do registro e armazenamento da avaliação de cada criança para acompanhamento do profissional e desfecho.
**Telas de orientação complementar**		
**Tela de orientação complementar**	Esquema básico de tratamento.	Será disponibilizado um quadro com esquemas de fármacos e fluxogramas orientando cada conduta de tratamento.
**Tela de método diagnóstico complementar**	lavado gástrico, PCR, lavado bronco-alveolar, BAAR, *swab.*	Orientação quanto à execução e direcionamento de exames que auxiliem na tomada de decisão.
**Tela de eventos adversos**	Possíveis eventos adversos que ocorrem na criança durante o tratamento.	Auxiliar o profissional quanto aos eventos adversos que ocorrem durante o uso da medicação.
**Tela de fluxograma de conduta**	Fluxograma de conduta para investigação e avaliação de TB ativa e ILTB.	Direciona o profissional de acordo com os critérios para TB ativa ou ILTB.
**Manuais do Ministério da Saúde**	Manuais de recomendações para TB.	Profissional poderá ter acesso às condutas mais atualizadas.

*Fonte: adaptado de Sant’ Anna et al. (2006) e Ministério da Saúde (2019).*

Para avaliação de risco para TB, foi utilizado o instrumento recomendado pelo MS para escore de classificação de risco para apoio ao diagnóstico da TB pulmonar em crianças e adolescentes, dividido em quatro itens: quadro clínico-radiológico; contato de adulto com TB; prova tuberculínica; e estado nutricional. Cada resposta dada a esses itens é convertida em uma pontuação específica^(4)^.

Para interface de avaliação do Índice de Massa Corporal (IMC) da criança, foi utilizado o gráfico de percentil utilizado pelo MS, empregando as medidas de peso, altura, sexo e idade da criança, obtidas no exame físico, transformadas em algoritmos e traduzidos para a linguagem do software^(25)^.

Na definição do *designer* do protótipo, os ícones escolhidos são representações visuais de conceitos-chave relacionados ao aplicativo, como criança, radiografia, pulmão, gotas e gráficos. Esses ícones foram selecionados para comunicar de forma direta o objetivo do aplicativo, que é auxiliar na identificação e diagnóstico precoce da TB em crianças, utilizando tecnologia de predição e apoio clínico.

Na etapa de elaboração, foi criado fluxo de informação para o direcionamento e definição dos atores que irão interagir com os sistemas e as funções do *TB Kids,* a fim de alinhar a programação do mundo real com a linguagem computacional utilizando diagrama de caso de uso e seus requisitos de interface com o usuário.

Utilizando o *scrum* para o gerenciamento do projeto, foram elaborados os *mockups* para a organização e compreensão da dinâmica do aplicativo. Portanto, esta etapa reúne o *front-end, back-end,* treinamento da IA e produção das telas com suas interfaces.

No desenvolvimento do *front-end,* houve a concatenação proposta pelo *designer* e sua adaptação com as funções destinadas à resposta do usuário, através de cálculos e representações abstratas de variáveis que são enviadas posteriormente ao servidor e banco de dados, como também folhas de estilos visuais que definem as diretrizes gráficas em listas de definições que unem valores, medidas, tamanhos, cores e demais parâmetros estéticos. Nesta fase, a experiência de usuário foi analisada através de testes internos em diferentes tipos de aparelhos celulares.

No desenvolvimento do *back-end,* foram definidos as tabelas e métodos de comunicação entre usuário e servidor, otimizando ao máximo o uso de dados e organizando as informações para serem acessadas em consultas por usuários cadastrados no sistema e por análises realizadas com potenciais pacientes. O diagrama de linguagem de modelagem unificada traçou a dinâmica e o fluxo do *software,* descrevendo as suas funcionalidades e conteúdo.

O treinamento das imagens utilizando IA seguiu os critérios de filtragem, edição e utilização das imagens de radiografias de tórax, a fim de categorizar e alimentar a plataforma de treinamento neural da IA. Paralelamente, foi gerada solução dinâmica e local (*offline*) para utilização dos resultados obtidos pela IA, por meio da exportação de bibliotecas orientadas ao uso em linguagem *Javascript,* projetadas para serem facilmente atualizadas e integradas em navegadores de internet e aplicativos. Foi utilizado o banco de dados públicos de imagem digital padrão para TB, criado pela Biblioteca Nacional de Medicina em colaboração com o Departamento de Saúde e Serviços Humanos, Condado de Montgomery, Maryland, EUA, disponível em https://openi.nlm.nih.gov/faq#collection/https://nihcc.app.box.com/v/Ches-tXray-NIHCC. A amostra dessa primeira etapa contou com 799 imagens de radiografias de tórax, tratadas e categorizadas como normais e anormais, com manifestações de TB, sendo 138 do banco de imagens de Montgomey (EUA) (80 imagens de radiografias de tórax normais/sem alterações pulmonares e 58 casos de imagens radiológicas com manifestações para TB) e 662 do banco de imagens da China (326 casos de radiografias normais/sem alterações pulmonares e 336 casos indicando alterações radiológicas para TB)^(26)^.

Para o treinamento da IA do protótipo *TB Kids,* foi escolhida a plataforma *Teachable Machine,* integrada ao restante do sistema através da importação do modelo treinado no formato TM, utilizando a tecnologia *TensorFlow* para *javaScript. TensorFlow* é uma biblioteca de código aberto para computação numérica e aprendizado de máquina em grande escala. O *TensorFlow* foi responsável por agrupar uma série de modelos e algoritmos de aprendizado de máquina e aprendizado profundo (redes neurais). Todos os algoritmos relacionados à predição de imagens e aprendizado de máquina estão relacionados ao *Tensorflow* e à plataforma *Teachable Machine* ^(27)^.

O passo inicial para o treinamento ser iniciado foi a padronização das imagens coletadas, eliminando todo resíduo textual das imagens e formatando-as em uma proporção 1:1 (um para um) de 224px/224px. O segundo passo foi a elaboração de categorias na plataforma de treinamento, para que a IA pudesse, através de suas redes neurais, aprender a diferenciar pulmões saudáveis de pulmões com TB (Figura 1). O terceiro passo foi o treinamento, exportação do modelo e implementação da conexão dinâmica na plataforma *Teachable Machine.*

**Figura 1:** Categorização das imagens para treinamento da Inteligência Artificial

Uma vez treinada a IA, o sistema precisava de integração com interfaces gráficas. O quarto passo foi a adequação do modelo a uma estrutura de dados que permitisse validação e teste de resultados. Utilizando a plataforma VSCode e as linguagens *Javascript,* css, php e html, foi elaborada uma aplicação *web* capaz de utilizar o modelo e analisar os resultados a partir da comparação com o aprendizado de máquina prévio. Todas as radiografias foram devidamente divididas, tratadas e categorizadas antes de serem utilizadas no treinamento.

A etapa de construção consiste na apresentação do protótipo *TB Kids,* o qual poderá ser acessado por meio de *link.* Para acesso, é necessário realizar cadastro e criar senha, que será armazenada de forma criptografada no banco de dados. Internamente, as aplicações compartilham o mesmo banco de dados, permitindo, assim, a interoperabilidade e a integridade dos dados.

A tela *Splash Screen* do *TB Kids* traz informações sobre o aplicativo, tais como o objetivo do *software,* população-alvo, como ele será utilizado, dando as boas-vindas ao profissional que irá acessá-lo (Figura 2).

**Figura 2:** Telas do protótipo *TB Kids*

Na Figura 2, na segunda tela (painel de controle), o usuário terá acesso às quatro funcionalidades principais do aplicativo. Após navegar pelas telas 1 a 5 de escore de risco, respondendo às questões específicas, o profissional terá a opção de anexar a imagem ou apontar a câmera para a radiografia que gostaria de realizar a leitura. O aplicativo irá iniciar a análise e gerar um resultado de diagnóstico da imagem e, caso identifique anormalidades, irá indicar alteração pulmonar detectada como resultado.

Ainda, mostra também a tela de resultado do escore gerado de acordo com a pontuação do grau de risco que a criança possui para TB. A interpretação segue o manual do MS: pontuação acima ou igual ≥ a 40 pontos: diagnóstico muito provável (iniciar tratamento); 30 a 35 pontos: diagnóstico possível (iniciar tratamento a critério médico); ≤ a 25 pontos: diagnóstico pouco provável (investigar e utilizar outros métodos)^(4)^.

O *TB Kids* apresenta também a opção de orientação complementar de possíveis condutas conforme o resultado: escore alto - iniciar o tratamento e terá disponível ao profissional o esquema básico de tratamento em crianças de acordo com o seu peso e idade (< de 10 anos de idade), composto por três fármacos na fase intensiva (RHZ) e dois na fase de manutenção (RH), com apresentações farmacológicas individualizadas (comprimidos e/ou suspensão); escore baixo - será orientado a outras opções de exames para investigação (lavado gástrico, PCR, lavado bronco-alveolar, BAAR, swab), e cada exame terá vídeos orientando o profissional na sua execução. Também são acessíveis orientações para possíveis eventos adversos durante o tratamento e algoritmos de diagnóstico de ILTB, compreendendo a importância de uma avaliação integral e efetiva para a TB nessa faixa etária.

A avaliação resultou em sete heurísticas violadas das dez propostas por Nielsen^(24)^, e foram identificados 38 problemas nas 22 telas do protótipo *TB Kids,* descritos na Tabela 1.

**Tabela 1:** Heurísticas violadas, problemas de usabilidade e as severidades encontradas nas telas do protótipo, Manaus, Amazonas, Brasil, 2023

Heurística violada	Problemas de usabilidade (porcentagem)	Severidades					Total
		0	1	2	3	4	
H1- Visibilidade do estado do sistema	7 (18,42%)			7			7
H2- Correspondência entre a interface do sistema e o mundo real	2 (5,26%)					2	2
H3- Controle e liberdade do usuário	7 (18,42%)				1		1
H4- Consistência e padrão	10 (26,32%)		1				1
H5- Prevenção de erros	2 (5,26%)		1	1			2
H7- Eficiência e flexibilidade de uso	2 (5,26%)		2				2
H8- Estética e *design* minimalistas	8 (21,05%)		8				8
Total por heurística	38 (100%)		12	8	1	2	38

Os resultados demostraram que “consistência e padrão” e “estética e *design* minimalistas” foram as heurísticas mais violadas, com dez (26,32%) e oito (21, 05%) problemas de usabilidade, seguidas por “visibilidade do estado do sistema” e “controle e liberdade do usuário”, com sete (18,42%) itens violados em cada heurística avaliada.

O protótipo foi testado por quatro avaliadores, e seus principais comentários fazendo uso das heurísticas foram: necessidade de melhoria do *design,* com alteração da paleta de cores do protótipo; as telas estavam poluídas e escuras, sem seguir padrões; máscara da data de nascimento ausente; necessidade de inserir botões de avançar e voltar nas telas, permitindo ao usuário controle de suas ações; houve também ausência de etapas sinalizadas nas telas de escore de risco e campos preenchidos nos botões nas telas de avaliação de PPD, necessitando serem desmarcados pelo profissional.

O problema de usabilidade que recebeu o maior grau de se-veridade^(4)^ foi a heurística “correspondência entre a interface do sistema e o mundo real”, indicando que o protótipo não gerava a pontuação correta no escore de risco e, consequentemente, interferia no resultado do diagnóstico. Outro item avaliado nesse mesmo grau foi a imprecisão ao gerar resultados na tela de avaliação do cálculo de IMC da criança; o erro foi corrigido imediatamente em ambos os itens, e foram realizados os retestes pelos avaliadores.

Quantos às correções do protótipo, foram realizadas mudanças no *design,* com alterações das cores do protótipo, tornando a tecnologia mais intuitiva e com menos poluição de conteúdo, organização dos conteúdos por tópico em uma linguagem de fácil compreensão. Foram inseridas explicações sobre o gráfico, e na tela inicial, o protótipo possuía informações de uso. Após a correção do *bug,* obteve-se a pontuação correta em cada item avaliado, gerando resultado compatível. Foram também adicionados os botões de avançar e voltar, sinalizados e centralizados em cada tela. Quanto ao cálculo de IMC, houve ajuste conforme o cálculo de percentil de acordo com o MS, sendo atualizado e testado, e para a tela de avaliação de PPD, foram removidos os campos pré-demarcados, sendo ajustados os campos para preenchimento.

## DISCUSSÃO

Sem tratamento, a taxa de mortalidade da TB é elevada (50%), enquanto a taxa de cura com os tratamentos recomendados pela OMS é de cerca de 85%, o que demonstra a importância do acesso ao diagnóstico de forma precoce, aliado a ações multissetoriais sobre fatores determinantes da TB, como pobreza, subnutrição, infeção pelo VIH, tabagismo e diabetes^(2)^. O conhecimento dos prestadores de cuidados de saúde sobre o rastreio e a detecção de casos de TB infantil é determinante para as melhores atitudes e práticas, e efetividade da investigação de contatos nas unidades de saúde e comunidade, o que tem relação com a disponibilidade de recursos e oferta de formação específica^(9)^.

São reconhecidos o protagonismo da enfermagem no en-frentamento da TB e a importância da informação tecnicamente fundamentada para ampliar o potencial das experiências no cotidiano dos serviços, em especial quando essas ferramentas fortalecem a autonomia do profissional em seu trabalho, são de fácil aplicabilidade e são produzidas de forma participativa^(27)^. Com um treinamento adequado, o aplicativo *TB Kids* pode se tornar uma ferramenta valiosa para apoio ao diagnóstico desse profissional na luta contra a doença em crianças na região amazônica e em outras áreas afetadas pela TB. Entre os estudos desenvolvidos utilizando imagens para alimentação de IA, ainda é desafiante ter um banco de imagens de radiografias torácicas de crianças com diagnóstico para TB^(15, 16)^.

A atualização da Resolução n° 736, de 17 de janeiro de 2024, pelo Conselho Federal de Enfermagem a respeito do processo de enfermagem prevê por parte do enfermeiro a autonomia na avaliação por meio de exames de imagem, sendo um suporte importante para condutas, desfechos e diagnósticos de enfermagem para esse profissional^(28)^.

A tecnologia *TB Kids* pode ser uma ferramenta crucial de apoio na interpretação e no julgamento clínico do enfermeiro, por ser direcionada a cuidados ordenados e à tomada de decisão mais assertiva de um paciente em risco para TB. Ao oferecer suporte sensível na identificação de alterações radiológicas expressivas para a doença, promove um apoio ao diagnóstico de forma sistemática e criteriosa, permitindo mensurar (pontuações) as chances de uma criança ser acometida pela TB, propor a melhor conduta terapêutica possível para essa população e diminuir os riscos potenciais de agravo e de diagnóstico tardio.

Os recursos educacionais e materiais de apoio adicionados ao aplicativo *TB Kids* visam fortalecer o conhecimento e a capacitação de enfermeiros e profissionais de saúde no enfrentamento da TB em crianças. Ao fornecer essas informações, o aplicativo busca melhorar a qualidade do atendimento e reduzir as fragilidades enfrentadas pelos profissionais no cuidado a crianças com diagnóstico presuntivo de TB.

Outro diferencial da tecnologia é a possibilidade de o enfermeiro ser direcionado também a condutas de investigação diagnóstica para a forma latente da doença. O profissional será direcionado a algoritmos e fluxogramas de investigação tanto para TB ativa quanto para ILTB, salientando a importância de tecnologias completas e que abordem todas as formas da doença, uma vez que a detecção da forma latente é um fator decisivo para interrupção da cadeia de trasmissão e eliminação da TB^(29)^.

O *TB Kids,* ao fornecer uma ferramenta tecnológica avançada como apoio ao diagnóstico da TB em crianças, poderá contribuir para a eliminação da TB e colaborar diretamente para efetivação das metas dos ODS. A eliminação da TB requer um compromisso renovado com a equidade, a justiça social e o uso de tecnologias inovadoras. A integração desses elementos em políticas de saúde pública e em programas específicos, como o *TB Kids,* pode não apenas acelerar o progresso em direção ao ODS 3, mas também contribuir para um desenvolvimento sustentável e inclusivo.

É importante considerar, durante o desenvolvimento de aplicativos móveis para o apoio ao diagnóstico, as recentes mudanças na Lei Geral de Proteção de Dados (LGPD), com o estabelecimento de regras claras sobre tratamento de dados pessoais e padronização de normas. Quanto às crianças, a LGPD diz que é necessário, para tratar os dados pessoais da criança, o consentimento específico dos responsáveis^(30)^.

Cabe ressaltar que a abordagem de treinamento com um conjunto diversificado e representativo de imagens é crucial para garantir a precisão e a confiabilidade dos resultados obtidos pelo aplicativo *TB Kids.* A qualidade e a quantidade de imagens são fatores determinantes para o desempenho do sistema de IA.

Ainda, o teste de usabilidade realizado confere um nível de *expertise* intrínseco ao processo, garantindo uma análise aprofundada e especializada do sistema. A utilização do *checklist* estruturado e da classificação de severidade de Nielsen oferece uma metodologia sistemática e rigorosa para a identificação, registro e priorização de falhas, proporcionando uma base sólida para a implementação de melhorias substanciais no sistema avaliado^(24)^.

A utilização de ferramentas como o *TB Kids* se alinha diretamente com o ODS 3 da ONU, que visa assegurar uma vida saudável e promover o bem-estar para todos, em todas as idades. O *TB Kids* não apenas apoia os profissionais de saúde no diagnóstico e tratamento da TB infantil, mas também contribui para a redução das disparidades na saúde e o fortalecimento dos sistemas de saúde.

### Limitações do estudo

Uma limitação do estudo foi o fato de a etapa de transição não ser contemplada. Em momento futuro, pretende-se realizar o treinamento dos usuários e o registro do produto na Agência de Inovação e Tecnologia da UEA, para posterior disponibilização nas lojas virtuais. Portanto, para as etapas de avaliação de confiabilidade e desempenho do aplicativo, espera-se realizar a coleta de radiografias de crianças de maneira prospectiva em unidades de referências no Estado.

### Contribuições para as áreas da enfermagem, saúde ou políticas públicas

O protótipo desenvolvido é uma ferramenta criativa e inovadora que responde à atual carência de ferramentas disponíveis que utilizam IA para diagnóstico de crianças com potencial risco para TB, representando, assim, uma importante contribuição para o enfrentamento da TB pulmonar infantil na Atenção Primária à Saúde. Essa tecnologia traz benefícios, como uma considerável redução do tempo de diagnóstico e aumento na precisão das avaliações clínicas, facilitando a identificação precoce de casos de TB em crianças. Além disso, ao integrar funcionalidades de IA, o protótipo apoia os profissionais de enfermagem na identificação e no monitoramento contínuo dos pacientes, contribuindo para seguimento adequado e manejo eficaz da doença. Dessa forma, ele fortalece as práticas de enfermagem e melhora a qualidade do cuidado oferecido na Atenção Primária à Saúde.

## CONCLUSÕES

O protótipo *TB Kids* é uma proposta transformadora para atuação da enfermagem no controle da TB infantil, já que tecnologias que utilizam IA para apoio ao diagnóstico da TB para essa população são restritas e a maior amplitude de atuação do enfermeiro neste campo precisa ser consolidada. Além da avaliação para TB ativa, tem como diferencial a possibilidade de investigação para a forma latente da doença como uma ferramenta completa e dinâmica que oferece ao profissional uma aplicação para continuidade e qualidade da assistência. Outro avanço que a tecnologia carrega é a de propor aos profissionais o acesso a materiais educativos para capacitação e aperfeiçoamento do conhecimento, sabendo que tecnologias com suporte educacional são dispositivos fundamentais para a qualidade do cuidado, além de foco privilegiado da intervenção de enfermagem.

Conclui-se que o protótipo *TB Kids* representa um avanço significativo no enfrentamento da TB pulmonar infantil. A escassez de ferramentas digitais de apoio ao diagnóstico para crianças torna o *TB Kids* uma ferramenta inovadora e potencializadora no contexto da saúde pública. Contudo, validar ferramentas inteligentes para uso em cenários reais é desafiador, visto que persiste como limitação a disponibilidade de bancos de imagens acurados para essa faixa etária. Dessa maneira, mais estudos se fazem necessários, e as contribuições deste produto tecnológico não pretendem substituir especialistas humanos.
